# Supplementary material for: Validity and precision of the International Physical Activity Questionnaire for climacteric women using computational intelligence techniques
Source: PLoS One. 2021 Jan 14;16(1):e0245240. doi: 10.1371/journal.pone.0245240 (PMC7808655; doi:10.1371/journal.pone.0245240)
Supplement: Attachment — Submitted filename: Response to Reviewers.doc [file pone.0245240.s002.doc]

Dear Dr. Seyedali Mirjalili

Academic Editor, Plos One Magazine

We initially record our thanks to the considerations on the article PONE-D-20-28544, entitled "Validity and precision of the international physical activity questionnaire for climacteric women using computational intelligence techniques" and to the suggestions presented. Below, we present our responses to each of the items scored, as a way to facilitate the review of the same and we are available for any clarifications or suggestions for improving the work.

Reviewer 1

| **Comments** | **Replies** |
| --- | --- |
| 1. Lack of literature review, the authors are suggested to discuss more recent and related works. | A literature review was carried out on the use of computational intelligence techniques for the selection of variables in epidemiological studies and for the validation of data collection instruments, which justifies the use of computational intelligence techniques for the validation of IPAQ for women weather, which was included in the introduction of the manuscript section. |
| 2. The main contributions of this paper is not clear. Please point out the main contributions in the last paragraph of Introduction. | As the last paragraph of the introduction of the manuscript, as suggested by the reviewer, we include the main contributions of this work. |
| 3. Authors are encouraged to provide a flow-diagram of the proposed work. | We created a flowchart (Figure 1), which was inserted in the introduction of the manuscript, showing all stages of validation and evaluation of the accuracy of the IPAQ. |
| 4. Random forest is a well regard classifier. The authors are suggested to apply it not only in measuring the important of the variable but also in classification stage. | Suggestion accepted. Random forest was used to classify the variables that make up the IPAQ. It was included in the classification methodology and results. |
| 5. The parameter settings of machine learning algorithms such as number of splits in DT, number of neurons, hidden layers in ANN, the kernel function used in SVM need to be provided. | The machine learning algorithm parameter settings, such as number of divisions in DT, number of neurons, hidden layers in ANN, the kernel function used in SVM were provided in the work methodology (Table 1), as suggested. |
| 6. Instead of using machine learning algorithms, deep learning methods such as convolutional neural network can be also applied for classification. | Yes, we agree with the reviewer, however, deep Convolutional RNAs need a large volume of characteristics and training examples to be efficient [1,2] which can be provided by complex data such as images, texts and videos and, therefore, are more indicated for these types of data. The data set used in this study has only 8 characteristics and 873 examples and, therefore, we consider it small for learning via Convolutional RNA. In addition, the purpose of using the Machine Learning methods for classification was to verify whether the elimination of variables 1 and 8 would affect the structure of the IPAQ and for that we used classification techniques widely used in the literature.  [1] RGB-D object recognition and pose estimation based on characteristics of pre-trained convolutional neural networks  [2] SALAMON, Justin; BELLO, Juan Pablo. Deep convolutional neural networks and increased data for classification of environmental sounds. IEEE Signal Processing Letters, v. 24, n. 3, p. 279-283, 2017. |
| 7. Authors should perform the statistical analysis (e.g. Friedman test or Anova test or Wilcoxon test) to support the classification results. | We agree with the reviewer. The Kruskal-Wallis test was used to compare the results. The data from the statistical test of the cross-validation is in the table referring to the cross-validation (Table 5). The statistical test data of everyone trained and everyone tested cannot be done because there is only a single execution of the algorithm for each configuration. |

Reviewer 2

| **Comments** | **Replies** |
| --- | --- |
| 1. The authors apply Kohonen and k-means algorithms to evaluate the validity of the (IPAQ) for climacteric women, why these algorithms, why not other algorithms? what is your contribution in terms of these computational intelligence? | KMeans was used because it is a popular method of data partitioning widely used in many fields, including data mining, pattern recognition, decision support and machine learning and also due to its ability to handle numerical variables and usage of a spacing heuristic to choose the initial groups in order to avoid suboptimal solutions. Kohonen RNA was used to reinforce kMeans' findings regarding optimal grouping, since both are partition based grouping methods but with different grouping heruistics. The two methods are quite consolidated in the architecture and meet the first objective of the article, which is to verify the number of categories that IPAQ is divided into. We have included this information in the manuscript methodology. |
| 2. The authors did not include related work of this study, existing methods and recent related work should be included and explored. | A literature review was carried out on the use of computational intelligence techniques for the selection of variables in epidemiological studies and for the validation of data collection instruments, which justifies the use of computational intelligence techniques for the validation of IPAQ for women weather, which was included in the introduction of the manuscript. |
| 3. For the method, authors should discussed and clearly explain the algorithms they used and show their contribution. | The machine learning algorithm parameter settings, such as number of divisions in DT, number of neurons, hidden layers in ANN, the kernel function used in SVM were provided in the work methodology, as suggested (Table 1). |
| 4. Another concern is that the figures and tables should be placed within the text. | Figures and tables have been included in the body of the text, as recommended. |
| 5. There is no statistical test to judge about the significance of the method’s results. Without such a statistical test, the conclusion cannot be supported. | We agree with the reviewer. The Kruskal-Wallis test was used to compare the results. The data from the statistical test of the cross-validation is in the table referring to the cross-validation (Table 5). The statistical test data of everyone trained and everyone tested cannot be done because there is only a single execution of the algorithm for each configuration. |
