# Supplementary material for: Validity and precision of the International Physical Activity Questionnaire for climacteric women using computational intelligence techniques
Source: PLoS One. 2021 Jan 14;16(1):e0245240. doi: 10.1371/journal.pone.0245240 (PMC7808655; doi:10.1371/journal.pone.0245240)
Supplement: Attachment — Submitted filename: Response to Reviewers 2.doc [file pone.0245240.s003.doc]

Dear Dr. Seyedali Mirjalili

Academic Editor, Plos One Magazine

We initially record our thanks to the considerations on the article PONE-D-20-28544, entitled "Validity and precision of the international physical activity questionnaire for climacteric women using computational intelligence techniques" and to the suggestions presented. Below, we present our responses to each of the items scored, as a way to facilitate the review of the same and we are available for any clarifications or suggestions for improving the work. We are left with a little doubt with the question number 4 of the reviewer 2, about the numbering of the sessions. We did not find this information in the magazine's formatting guidelines, but as he suggested, we did so in the body of the text.

Reviewer 1

| **Comments** | **Replies** |
| --- | --- |
| 1. In Table 5, please provide the detail how the p-value is calculated. Also, the p-value was very high, a discussion on this finding is required. | Methods were included in the session as the p-value was calculated, and we inserted information about the p-value as a caption in Table 5. In this study we adopted a significance level of 0.05, that is, for values less than or equal to 0.05 we reject the null hypothesis (which says that among the groups evaluated all values are equal) and for values greater than 0.05 we accept the null hypothesis. That is, when the p-value is greater than 0.05 it means that all values are equal, therefore, for values considered low or high, the level of significance was respected. The Kruskal-Wallis test is a non-parametric method that uses the difference between the averages of the stations in each group to determine the p-value, in this study this difference is small for most cases of comparison and a small difference contributes to a high p-value. For example, in the precision of the SVM method, the posts in each group had averages of: 9, 10.9, 10 and 12.1 which generated a p-value of 0.859. The recall of the SVM had rank averages of: 9.9, 10.5, 10.6 and 11 and this generated a p-value of 0.993. |

Reviewer 2

| **Comments** | **Replies** |
| --- | --- |
| 1-Figure 1, the caption should be placed below the figure. Also, here as suggested in previous review to include the flowchart of the proposed method and the description of the proposed methods in methodology part. | We included a caption below the figure and inserted the flowchart in the methods section, as suggested by the reviewer. |
| 2- The authors recommended to reorganized the method, so they include first the proposed method mention in 1. then (Participants, Instruments, International Physical Activity Questionnaire (IPAQ) and Procedures) put all of them in one section named e.g. (Area of the study). | We accepted the reviewer's suggestion, and the participating sessions, instruments, International Physical Activity Questionnaire (IPAQ) and procedures were included in a single session called study procedures. |
| 3- The authors used Kruskal-Wallis Test which is good, however they didn't explain within the text which method is significant using this test. Include the discussion of the use of the Kruskal-Wallis Test. | Methods were included in the session as the p-value was calculated, and we inserted information about the p-value as a caption in Table 5. In this study we adopted a significance level of 0.05, that is, for values less than or equal to 0.05 we reject the null hypothesis (which says that among the groups evaluated all values are equal) and for values greater than 0.05 we accept the null hypothesis. That is, when the p-value is greater than 0.05 it means that all values are equal, therefore, for values considered low or high, the level of significance was respected. The Kruskal-Wallis test is a non-parametric method that uses the difference between the averages of the stations in each group to determine the p-value, in this study this difference is small for most cases of comparison and a small difference contributes to a high p-value. For example, in the precision of the SVM method, the posts in each group had averages of: 9, 10.9, 10 and 12.1 which generated a p-value of 0.859. The recall of the SVM had rank averages of: 9.9, 10.5, 10.6 and 11 and this generated a p-value of 0.993. |
| 4. Its recommended to follow the journal format and numbering of sections. | Suggestion accepted, we have numbered the sections of the article. |
